# Effects of *Polygonatum cyrtonema* flour addition on the food quality, microstructure, *in vitro* digestibility and antioxidant capacity of rice noodles

**DOI:** 10.1016/j.fochx.2025.102710

**Published:** 2025-07-03

**Authors:** Yongguang Zhu, Qianwei Cheng, Jiayan Zhang, Peng Chen, Yuan Cheng, Luli Meng, Jie Chen, Tong Chen

**Affiliations:** a School of Biological and Chemical Engineering, Guangxi University of Science and Technology, Guangxi Liuzhou Luosifen Center of Technology Innovation, Liuzhou 545006, PR China; b Guangxi Sanmenjiang Ecological Camellia Oil Co. Ltd., Liuzhou 545006, PR China

**Keywords:** Rice noodle, *Polygonatum cyrtonema* flour, Dietary fibre, Microstructure, *In vitro* digestibility, Antioxidant capacity

## Abstract

*Polygonatum cyrtonema* flour (PCF) is rich in active substances such as flavonoids, polysaccharides, and trace elements, which can be added to food to enhance its nutritional value while imparting product quality. In this study, we investigated the effects of PCF on the quality and characteristics of rice noodles. The addition of PCF to rice noodles conferred a yellow colour, while increasing cooking quality. Pasting characterization showed that the viscosity of the *P. cyrtonema* rice noodles (PCRN) was lower than the control group. Thermal characterization revealed that the thermal stability of the rice noodles increased with the addition of PCF. The ordered structure and microstructure of PCRN were enhanced under specific conditions of addition. Furthermore, the digestion resistance and antioxidant capacity of the noodles were enhanced. The results showed that the rice noodles produced by adding PCF had better quality, richer nutrition and better resistance to digestion than the control group.

## Highlights

• Polygonatum cyrtonema flour (PCF) can reduce the viscosity and effectively inhibit ageing of rice flours.
• PCF improves the relative crystallinity and enhances the short-range ordered structure of rice noodles.
• PCF can inhibit the digestion of rice noodles.
• PCF helps to form more holes and form a better protein starch cross-linking network in rice noodles.
• PCF increases the total phenol content and improves the antioxidant capacity in rice noodles.
• PCF can improve sensory evaluation scores and increase acceptance.

## 1 Introduction

Rice is a crucial cereal grain that provides a significant source of energy in the human diet and serves as a staple food for approximately 50 % of the global population (Karizaki, 2016; Liu et al., 2016). A diverse array of starch–based products made from rice is available, noted for their palatable taste and hypoallergenic characteristics, which are particularly beneficial for individuals with celiac disease. These rice–based alternatives can effectively replace wheat flour in various baked goods (Amin et al., 2017; Zhang et al., 2022; Zhang, Sun, & Ai, 2022). Rice noodles, products originating from rice, are particularly popular in Southern China and Southeast Asia, and their consumption has recently expanded to Europe and the United States (Wei et al., 2022). Refined rice, commonly referred to as white rice, serves as the primary raw material for rice noodle production and only retains the endosperm of the rice grain. Most nutrients, excluding carbohydrates, are concentrated in the bran, endosperm, dextrinoid layer, and embryo (often referred to as sugar layer) (Geng et al., 2020； Spaggiari, Dall'Asta, Galaverna, & Del Castillo Bilbao, 2021). Consequently, refined rice only contains a relatively single nutritional component, which does not conform to contemporary nutritional standards. Furthermore, rice is easily digestible and many rice–based foods exhibit a high glycaemic index. A prolonged diet characterised by high energy, low nutrient density, and elevated carbohydrate content is likely to contribute to the rising prevalence of obesity, as well as an increased risk of type II diabetes mellitus and cardiovascular diseases (Kan, Oliviero, Verkerk, Fogliano, & Capuano, 2020). Research indicates that the incorporation of brown rice or the addition of other ingredients into refined rice noodles can mitigate the challenges associated with the limited nutritional profiles and easy digestion of rice noodle products (Liu et al., 2024； Mo et al., 2024).

*Polygonatum cyrtonema* (PC) is a traditional Chinese medicine derived from the dried rhizomes of *Polygonatum kingianum* Coll. et Hemsl., *Polygonatum sibiricum Red*., and *Polygonatum cyrtonema Hua* all of which belong to the family Liliaceae (Wang et al., 2022). Contemporary studies have indicated that PC possesses significant properties, including antioxidant activity, reduction of blood glucose and lipid levels, the mitigation of fatigue and ageing, and immunomodulatory effects (Gu et al., 2020；Li et al., 2023; Li et al., 2023; Li et al., 2023). The therapeutic efficacy of PC is largely ascribed to its abundant active constituents, including polysaccharides, polyphenols, flavonoids, volatile compounds, alkaloids, and lignans, all of which demonstrate considerable pharmacological activity (Zhao et al., 2018). The nutritional composition of the PC also includes proteins and fats, and it serves as a significant source of potassium, calcium, magnesium, iron, and amino acids (Lu et al., 2024). Dietary fibre is a primary component of PC and provides various advantages to the human gastrointestinal system. It contributes to the enhancement of the intestinal microbiota, thereby aiding in disease prevention, facilitating intestinal tract cleansing, increasing satiety, and fulfilling other critical functions (Li et al., 2022; Li et al., 2022; Li, Ji, & Li, 2022). Moreover, dietary fibre can slow down starch digestion, which may be particularly effective in mitigating the risk of type 2 diabetes (Li et al., 2020). Research conducted by Zhang, Sun, and Ai (2022) indicated that dietary fibre can function as a physical barrier, thereby decreasing starch digestibility. Additionally, from both functional and technological perspectives, the integration of dietary fibre into food products can enhance their overall quality and affect their rheological, textural, and sensory characteristics, as well as prolong their shelf life, owing to their water–binding, gel–forming, organising, and thickening properties (Zafar et al., 2015). The incorporation of dietary fibre into starch–based food products has emerged as a prevalent trend in contemporary food science (Li, Ji, & Li, 2022; Li, Liu, et al., 2022; Li, Zhao, et al., 2022).

In response to the rising public health awareness, there has been a notable increase in the development of functional foods that utilise PC, such as PC–infused teas, beverages, sugars, and wines (Song et al., 2024). Concurrently, studies have begun to integrate PCF into starch–based food products. For example, Zheng et al. (2024) demonstrated that the incorporation of PCF into rice cakes not only modified their visual appearance, texture, and internal structure but also improved their resistance to digestion, mitigated the ageing process of yellow–essence rice cakes, and introduced novel flavours. Additionally, Xiong et al. (2023) produced cookies incorporating PCF, revealing that the addition of PCF enhanced the quality of the cookies by improving resistance to digestion and delaying starch retrogradation. Consequently, the present study investigated the addition of PCF to rice noodle, focusing on the edible quality, microstructural characteristics, antidigestive properties, and antioxidant capacities of PCRN. The objective of this study was to make a nutritious, digestion–retarding, and health–promoting rice noodle with a unique flavour profile. Furthermore, this study offers new insights and opportunities for advancing *P*. cyrtonema–based foods, which can enhance the value associated with PC.

## 2 Materials and methods

### 2.1 Materials

Japonica rice was supplied by Guangxi Fuxiang Grain and Oil Co Ltd. (Liuzhou, China; carbohydrate 86.60 %, protein 6.52 %, oil 0.45 %). PCF was supplied by Hangzhou YaMi Agricultural Science and Technology Co Ltd. (Hangzhou, China; dietary fibre 79.87 %, protein 9.11 %, oil 1.91 %). Amyloglucosidase (289653–100G, ≥100,000 units.g) was purchased from Beijing Huamaike Biotechnology Co. Pepsin (P6322–5g, >3000 U/mg), trypsin (T6325–5g, trypsin 1:250), α–amylase (A800731–500g, 35 U/mg), 1,1–diphenyl–2–trinitrophenylhydrazine (DPPH), 2,2–biazido–bis–(3–ethylbenzothiazoline–6–sulfonic acid) (ABTS) and Folin–Ciocalteu reagents were purchased from Shanghai Macklin Biochemical Technology Co. All chemicals were analytically pure except for methanol, which was chromatographic grade. All chemicals were procured from Xilong Chemical Co.

### 2.2 Preparation of compound powder

Rice was ground in a high speed mill (DFY–1000C, Linda Machinery, China) and the fraction that passed through a 100–mesh sieve is designated for use as rice flour. Compound powder was prepared by replacing rice flour with PCF in different percentages (5 %, 10 %, 15 % and 20 %) of rice flour. The control sample consisted of 100 % rice flour.

### 2.3 Pasting properties

According to the method of Hu, Chen, Zhao, Chen, and Wang (2020) with modifications, 3 g of the compound powder mixed in various ratios was weighed into the sample jar of a Rapid Viscometer Analyser (RVA–TecMaster, Perten Riva, Sweden), and then 25 mL of distilled water (14 % of the total water content) was added to the sample jar, which was then placed into the tester. The temperature changes were as follows: maintained 50 °C for 1 min, then rose to 95 °C within 3.75 min, held 95 °C for 2.5 min, subsequently decreased 50 °C within 3.75 min, and finally maintained 50 °C for 1.5 min. The speed was varied as follows: the stirrer speed was increased to 960 r/min within the first 10 s, and then maintained at 160 r/min. Viscosity was expressed in rapid viscosity units (cP).

### 2.4 Thermal properties

The compound powder (5.0 ± 0.20 mg) was weighed into an aluminium box with a lid, 8 μL of ultrapure water was added to the box, and the sample was equilibrated at 4 °C for 12 h. The temperature was increased from 20 to 100 °C in a differential scanning calorimeter (DSC3500, NETZSCH, Germany) at a rate of 10 °C.

### 2.5 Method of production of rice noodles

Different proportions of the compound powder and control sample were added to deionised water at a ratio of 60 % (*w*/*v*) and stirred for 1 min, the stirred rice paste was spread out and steamed in a pot for 15 min for maturation. Subsequently, the rice dough was kneaded for 3 min and formed into strips, placed into an extruder, extruded into strips, and heated again under the same heating conditions for 3 min for the second maturation. Then, it was taken out and rinsed with cold water to obtain *P. cyrtonema* rice noodle (PCRN).

### 2.6 Cooking quality

Approximately 20 cm or more of PCRN (35 g in total) was selected, placed in 300 mL of boiling water, immersed for 3 min, then fixed out and drained in cold water. The weights of the samples with a length of 10 cm or less were recorded as M_1_, and the weights of those with a length of 10 cm or more were recorded as M_2_. Moreover, PCRN sample with a mass of M_3_ was also weighed, then placed in boiling water (200 mL) and boiled continuously for 3 min before being fished out. Its then the surface was then rinsed with distilled water (50 mL). The rinsing and boiling solutions were mixed and further heated; after the water had largely evaporated, the residue was dried in an oven (105 °C) to constant weight, and the weight of the solids was recorded as M_4_. The water content of PCRN was determined as W using the constant–weight method. Eqs. (1), (2) were used to calculate the breakage rate and cooking loss, respectively.

(1) $$B\text{reakage}\;R\mathrm{ate}\;\left(\%\right)=\frac{M_{1}}{M_{1}+M_{2}}\times 100$$

(2) $$C\text{ooking}\;L\mathrm{ose}\;\left(\%\right)=\frac{M_{4}}{M_{3}\left(1‐W\right)}\times 100$$

### 2.7 Texture analysis

The textural properties of the rice noodles were determined using a texture tester (CT3–100 TPA, BROOKFIELD, USA) in TPA mode with a TA11/1000 probe. The test parameters were as follows: test speed, 0.5 mm/s; trigger force, 6 g; distance, 1 mm. Hardness, cohesiveness, springiness, gumminess, and chewiness parameters were determined. Each sample was evaluated at least five times, and the results were averaged.

### 2.8 Colour measurement

Freeze–dried PCRN was ground through a 100–mesh sieve to produce a dried powder. Approximately 3 g of dried powder was placed on white paper, and its colour was assessed using a handheld colourimeter (LS131, Linshang Technology, China). The L^⁎^, a^⁎^ and b^⁎^ values were recorded each time.

### 2.9 X–ray diffraction (XRD) analysis

Determinations were performed using an X–ray diffractometer (D/MAX–3BXX, Rigaku, Japan). The dried powder was placed flat on a support table, and Cu–Kα radiation (λ = 0.1543 nm) was applied with a test voltage of 40 kV, a current of 40 mA, a scanning diffraction angle of 5–40° and a scanning rate of 2°/min. The relative crystallinity (RC) was then calculated using Origin software.

### 2.10 Fourier transform infrared (FT–IR) spectroscopy analysis

FT–IR spectroscopy was performed on the samples according to the method described by Yang, Dong, Fang, Li, and Li (2024). The sample was placed on an ATR attachment spectrometer (Frontier/Nicolet 380, Perkin–Elmer, USA). 32 scans were performed at room temperature with a resolution of 4 cm^−1^ and a scan range of 400–4000 cm^−1^ using air as background. The IR spectrograms were deconvoluted to improve the resolution using OMNIC 9.0; and the peaks at 1047, 1022, and 995 cm^−1^ were extracted and their ratios were calculated.

### 2.11 Scanning electron microscopy (SEM) analysis

The prepared PCRN was quickly frozen at −80 °C in a freezer for 6 h. It was then transferred to a vacuum freeze dryer for 48 h. Freeze–dried rice noodle sections were sprayed with gold and imaged using a SEM (SU8010; Hitachi, Japan) for scanning and photographing. The scanning electron microscope was set at an accelerating voltage of 5 kV and magnifications of 800× and 1500×.

### 2.12 Confocal laser scanning microscopical (CLSM) analysis

PCRN was cut into 1 mm thin slices, placed on microscope slides, and infiltrated with 0.2 % (*w*/w) fluorescein 5–isothiocyanate (FITC) stain and 0.02 % (w/w) Rhodamine B stain for 10 min. After staining, the slides were rinsed with a small amount of distilled water, and placed on a carrier stage. Staining was observed using a laser confocal microscope (Nikon A1R; Nikon, Japan). The excitation wavelengths for FITC and Rhodamine B were 488 nm and 561 nm, respectively.

### 2.13 *In vitro* digestion of different rice noodles

#### 2.13.1 Hydrolysis curves of different rice noodles *in vitro*

The analysis was conducted using the method of Englyst, Kingman, and Cummings (1992) with some modifications. The dried powder (0.5 g) was placed in a 50 mL centrifuge tube and mixed well with 5 mL HCl–KCl buffer solution (0.01 M, pH 1.5) containing pepsin. The mixed solution was shaken for 30 min at 37 °C. Then, 5 mL of 0.01 M NaOH and 15 mL of 0.1 M sodium acetate buffer (pH 6.8) were added successively to the test tube and mixed well. Subsequently, 1 mL of a 1 mg/mL trypsin solution, 5 mL of a 3000 U/mL amyloglucosidase solution, and 0.1 mL of a 250 U/mL porcine pancreatic α–amylase were added to the mixture, which was then shaken at a constant temperature of 37 °C for 3 h. Aliquots were then taken at 0, 10, 20, 30, 60, 90, and 120 min. An aliquot (1 mL) of the hydrolysis solution was taken at 180 min, and anhydrous ethanol (4 mL) was added to inactivate the enzyme. After centrifugation (5000*g*, 5 min), the glucose content of the supernatant was determined using the 3,5–dinitrosalicylic acid (DNS) colourimetric method. The glucose content in the mixture was measured using an ultraviolet (UV) spectrophotometer (TU–1950, Pulsar, China), and the reducing sugar content of the *in vitro* digest of rice noodle was calculated, The starch digestibility rate was calculated according to Eq. (3).

(3) $$\text{Starch desgestibility rate}\;\left(\%\right)=\frac{\text{Content of hydrolyzed}\;\text{glucose}\times 0.9}{\text{Total starch weight}}$$

#### 2.13.2 Determination of rapidly digestible starch (RDS), slowly digestible starch (SDS), and resistant starch (RS) content

Referring to Englyst et al. (1992) and Englyst, Veenstra, and Hudson (1996), starches were classified as RDS, SDS or RS, according to the rate of hydrolysis in the gastrointestinal tract. RDS, SDS, and RS are the fractions digested within 20 min, digested between 20 and 120 min, and undigested after 180 min, respectively. Therefore, based on the hydrolysis data in Section 2.13.1. above, the RDS, SDS and RS contents were calculated using Eqs. (4), (5) and (6).

(4) $$\mathrm{RDS}\;\left(\%\right)=\frac{\left(\mathrm{Glu}\cos e_{20}-\mathrm{FG}\right)\times 0.9}{\mathrm{Total starch weight}}$$

(5) $$\mathrm{SDS}\;\left(\%\right)=\frac{\left(\mathrm{Glu}\cos e_{120}-\mathrm{Glu}\cos e_{20}\right)\times 0.9}{\mathrm{Total starch weight}}$$

(6) $$\mathrm{RS}\;\left(\%\right)=100-\mathrm{RDS}-\mathrm{SDS}$$

### 2.14 Total phenolic content (TPC) and antioxidant capacity

Dried powder from each sample (1.00 g) was mixed with 30 mL of methanol, shaken at a constant temperature of 30 °C for 2 h, and then centrifuged at 4000 rpm for 10 min. The supernatant was used as the extract for measuring TPC, DPPH and ABTS tests.

TPC was determined using the Folin–Ciocalteu method. After adding 1 mL of the supernatant, 1 mL of Folin–Ciocalteu reagent was added, followed by 3 mL of 7.5 % Na_2_CO_3_ solution. The mixture was stored in the dark for 1 h. The absorbance was measured at a wavelength of 760 nm and then substituted into the standard curve equation to calculate TPC. Gallic acid was used as the standard, and different concentrations of gallic acid standard solutions were prepared following the same protocol as that for the samples to determine the absorbance. The absorbance values were plotted as vertical coordinates, and the gallic acid concentrations were plotted as horizontal coordinates to generate a standard curve.

The DPPH solution was prepared using anhydrous ethanol. The supernatant was diluted 10 times as the test solution. Three millilitres of the test solution were placed in a test tube and thoroughly mixed with 4 mL of DPPH solution. The mixture was stored in the dark for 30 min and served as the test group. The absorbance of the test group was measured at 517 nm using a UV spectrophotometer (TU–1950, PERSEE, China) (denoted as A_1_). The blank group was prepared using 4 mL of anhydrous ethanol instead of DPPH solution, and its absorbance was denoted as A_2_. For the control group, 4 mL of DPPH solution was mixed directly with 3 mL of anhydrous ethanol, and the absorbance was recorded as A_3_. The decrease in absorbance of the reaction system indicated its ability to scavenge DPPH radicals. The DPPH scavenging rate was calculated using Eq. (7).

ABTS solution (10 mL, 7 mol/L) was mixed with 176 μL of potassium persulfate solution (7.35 mol/L) and stored in the dark for 12–16 h to form ABTS+, which was recorded as ABTS+ mother liquor. The ABTS+ mother liquor was adjusted with anhydrous ethanol to give an absorbance of (0.70 ± 0.02) at a wavelength of 734 nm and recorded as the ABTS+ working solution. The ABTS+ working solution (4 mL) was mixed with 0.2 mL of the sample solution, forming the test group, and the absorbance was measured after 6 min of reaction in the dark (denoted as A_4_). The sample solution was replaced with anhydrous ethanol as a control group and the absorbance was denoted as A_5_. The ABTS radical scavenging rate was calculated using Eq. (8).

(7) $$Ra\mathrm{dical}\;\mathrm{scavenging rate}\;\left(\%\right)=\frac{A_{1}-A_{2}}{A_{3}}\times 100$$

(8) $$Ra\mathrm{dical}\;\mathrm{scavenging rate}\;\left(\%\right)=\left(1-\frac{A_{4}}{A_{5}}\right)\times 100$$

### 2.15 Sensory evolution

Sensory evaluation was performed following the methodology described by Yang et al. (2025), with minor modifications. The noodles were cooked and cooled for further analysis. A group of 10 trained laboratory members formed a sensory assessment team. The panellists were given water to rinse their mouths before evaluating each sample. The samples were placed individually in a sealed plastic cups and randomly presented to each panellist. The scores table used for the sensory evaluation are presented in Table S1, which includes aroma (0–15 points), surface structure (0–25 points), textural properties (0–45 points), and taste (0–15 points).

### 2.16 Statistical analysis

All experimental data were measured at least three times in parallel, and results were expressed as mean ± standard deviation. Statistical analyses were performed using SPSS 26.0; one–way analysis of variance (ANOVA) was performed using the ANOVA algorithm, and Origin 2021 was used to graph the data. The level of significance was set at *P* < 0.05 for all significant differences.

## 3 Results and discussion

### 3.1 Analysis of Pasting properties

Table 1 presents the pasting characteristics of the compound powders at various addition levels and the control group as analysed by the Rapid Viscosity Analyser (RVA). The peak viscosity (PV), through viscosity (TV), breakdown (BD), final viscosity (FV), and setback (SB) of the blended powders decreased with increasing levels of PCF compared to the control. This phenomenon may be attributed to the interaction between PCF dietary fibres and phenolic compounds with the starch molecules produced (Chen et al., 2019；Han et al., 2020).

**Table 1:** Pasting property and thermal property from PCRN in different proportions.

Properties	Control	5 % PCF	10 % PCF	15 % PCF	20 % PCF
Pasting property					
Peak Viscosity (cP)	3206.33 ± 8.96a	2612.33 ± 20.98b	2288.67 ± 27.15c	1994.00 ± 7.94d	1781.33 ± 17.01e
Trough Viscosity (cP)	2274.67 ± 15.82a	1883.00 ± 7.21b	1765.67 ± 15.63c	1603.67 ± 11.93d	1480.67 ± 18.61e
Breakdown (cP)	931.67 ± 24.58a	729.33 ± 27.39b	523.00 ± 17.00c	390.33 ± 19.40d	300.67 ± 2.08e
Final Viscosity (cP)	4219.67 ± 20.03a	3607.67 ± 13.43b	3321.67 ± 20.21c	2981.33 ± 7.64d	2705.33 ± 38.07e
Setback (cP)	1945.00 ± 20.42a	1724.67 ± 18.72b	1556.00 ± 12.29c	1377.67 ± 11.06d	1224.67 ± 19.50e
Thermal property					
Onset temperature (°C)	74.967 ± 0.461d	76.167 ± 0.058c	76.567 ± 0.040bc	77.300 ± 0.361b	78.500 ± 0.300a
Peak temperature (°C)	83.167 ± 0.152e	84.033 ± 0.115d	84.767 ± 0.153c	85.167 ± 0.178b	86.300 ± 0.361a
Conclusion temperature (°C)	90.533 ± 0.091d	91.267 ± 0.208 cd	92.067 ± 0.493c	92.133 ± 0.208b	93.233 ± 0.200a
ΔH (J/g)	3.070 ± 0.019c	3.441 ± 0.201b	3.647 ± 0.056a	3.598 ± 0.034a	3.212 ± 0.006c

The values represent means of triplicate ± standard deviation. Values in the same column with different letters are significantly different (P < 0.05).

PV is the maximum viscosity value, reflecting the ability of the sample particles to absorb water and swell before disintegration, as well as their capacity to bind water, which influences the quality of the final product (Loubes, González, & Tolaba, 2018). The incorporation of PCF limited the free–swelling capacity of starch granules. This limitation could be attributed to the dietary fibre present in PCF, which competes with starch granules for water molecules during the pasting process. Meanwhile, the addition of PCF led to the dilution of starch granules, thereby reducing their swelling capacity (Mahmood et al., 2017). Wang, Ma, Lv, Wu, and Tan (2025) demonstrated that dietary fibre inclusion reduces the amount of dextrinisable starch, while the hydrophilic components in the dietary fibre diminish the swelling of rice starch, thereby decreasing PV and FV. BD represents the difference between PV and TV, and its magnitude is utilised to characterise the degree of viscosity reduction when the starch viscosity reaches its maximum value and is subsequently heated and sheared. This measurement not only reflects the shear resistance of the starch system at elevated temperatures but also indicates the thermal stability of the starch system (Wang et al., 2018). A higher BD value suggests that the starch system exhibits poorer shear resistance and thermal stability. Table 1 shows that the BD decreased significantly (*P* < 0.05) with increasing PCF content, indicating that the incorporation of PCF progressively enhanced the shear resistance and thermal stability of the system. SB is defined as the difference between FV and TV and serves as an indicator of the extent of short–term starch retrogradation (Zhang et al., 2024). The higher the SB value, the higher the short–term regeneration degree of the starch gelatinization system during the cooling stage. As shown in Table 1, the SB of the PCRN significantly (P < 0.05) decreased. The same phenomenon was found in Liu et al. (2024)‘s research. The retrogradation inhibition effect of PCR may be attributed to its high content of dietary fibre, polyphenols, and flavonoids. Dietary fibres could establish a physical barrier among starch molecules, consequently delaying the process of starch recrystallization (Xu, Zhang, Zhang, & Tan, 2021). Meanwhile, phenolic and flavonoid compounds, as polyhydroxy compounds, interact with starch through hydrogen bonding and hydrophobic interactions, thereby hindering molecular reassociation between starch chains. This mechanism effectively delays the short-term retrogradation of starch (Huo et al., 2025； Zheng et al., 2020). Notably, although rice noodles with higher short-term retrogradation generally show greater hardness and chewiness, rice noodles with higher PCR content exhibited lower SB values but higher hardness. This phenomenon can be attributed to PCR incorporation, which inhibits starch retrogradation while concurrently introducing significant quantities of dietary fibre that impart matrix-level structural rigidity to rice noodles, thereby counteracting textural modifications associated with reduced retrogradation (Tian et al., 2025).

### 3.2 Analysis of thermal properties

Endothermic transition temperatures (including the onset temperature, peak temperature, and end temperature) and enthalpy change (ΔH) were identified as the primary parameters of interest in thermal characterization (Jin & Xu, 2020). Table 1 summarises the variations observed in the four thermal property indices. Following the blending process, the endothermic transition temperatures of the mixed powders increased, accompanied by a significant alteration in ΔH (*P* < 0.05). The endothermic transition temperature serves as an indicator of the ease of gelatinization within the starch system; a lower endothermic transition temperature implies that the starch system gelatinises more readily, which also suggests a less thermally stable structure (Wang et al., 2023). As shown in Table 1, the endothermic transition temperatures increased significantly (P < 0.05) after the incorporation of PCF. This observation indicates that the thermal stability of PCRN was significantly enhanced, enabling the starch system to exhibit improved resistance to thermal degradation. This enhancement was likely attributable to the dietary fibres present in the PCF, which contained a significant number of hydrophilic groups that competed with the starch molecules for water absorption, thereby increasing the endothermic transition temperature. Furthermore, the abundant polyphenolic flavonoids in PCF may have contributed to this effect by reinforcing the hydrogen bonding network within the starch system, resulting in a more stable starch structure and, consequently, an elevated endothermic transition temperature (Li, Jiang, et al., 2023; Li, Li, et al., 2023; Li, Shen, et al., 2023). The change in ΔH reflected the energy required to disrupt the double helix structure within the starch system (Sun, Wang, Ma, & Wang, 2020). In summary, a higher ΔH value indicates that a greater amount of energy is necessary to disrupt the starch structure. The results demonstrated that the addition of PCF significantly increased the ΔH value of rice noodles (P < 0.05), suggesting that PCRN required more energy to break down the starch structure. Prior research has established that, under specific conditions, the variation trend of ΔH exhibits a strong correlation with that of RC, which is consistent with the observations in our study. Consequently, the increase in ΔH values may be attributable to the development of an ordered starch structure and the elevation of RC within PCRN.

### 3.3 Analysis of cooking quality

Cooking quality was identified as a fundamental indicator of the overall quality of rice noodles. As shown in Table 2, compared to the control group, the breakage rate and cooking loss of rice noodles were significantly reduced. This improvement was likely attributable to the dietary fibres present in the PCF, which reinforced the spatial network structure of starch, enabling the rice noodles to maintain better structural integrity during the boiling process. However, when the PCF addition exceeded 10 %, the cooking loss of rice noodles began to increased. This phenomenon was potentially PCF incorporation within the rice noodle gel network structure, leading to microstructure reconstruction and incomplete gelatinization of some starch granules (Zheng et al., 2024). The thermal properties suggested that the difficulty of starch gelatinization increased with increasing levels of PCF incorporation. Under identical processing conditions, samples with elevated PCF contents exhibited a lower degree of gelatinization than those with lower PCF contents. Furthermore, microstructural analyses revealed that higher PCF levels had an adverse effect on the rice noodle structural integrity. Consequently, certain PCF particles and starch granules are dislodged from the network structure during the cooking process, contributing to an increase in breakage rate and cooking losses.

**Table 2:** Cooking quality, texture, colour, FTIR and *in vitro* digestive parameters of PCRN in different additions.

Properties	Control	5 % PCF	10 % PCF	15 % PCF	20 % PCF
Cooking quality					
Breakage rate (%)	5.16 ± 1.06a	2.84 ± 0.57b	0.83 ± 0.13d	1.33 ± 0.05c	1.63 ± 0.06c
Cooking Lose (%)	24.83 ± 0.76a	16.33 ± 0.41c	10.29 ± 0.22e	14.50 ± 0.40d	18.32 ± 0.30b
Texture					
Hardness (g)	617.00 ± 66.18c	683.00 ± 26.56bc	702.83 ± 57.65bc	763.00 ± 12.49b	816.00 ± 56.82a
Cohesiveness	0.74 ± 0.03a	0.66 ± 0.02b	0.62 ± 0.01c	0.57 ± 0.02d	0.52 ± 0.01e
Springiness (mm)	0.79 ± 0.04a	0.78 ± 0.10a	0.72 ± 0.07ab	0.69 ± 0.02ab	0.61 ± 0.01b
Gumminess (g)	452.00 ± 75.9c	466.67 ± 58.71c	483.67 ± 87.84c	533.00 ± 56.29b	647.33 ± 78.04a
Chewiness (mJ)	2.7 ± 0.62c	3.13 ± 0.29bc	3.73 ± 0.68ab	4.07 ± 0.38ab	4.57 ± 0.29a
Colour					
L^⁎^	97.43 ± 0.04e	77.93 ± 0.08d	69.71 ± 0.12c	61.52 ± 0.13b	58.59 ± 0.14a
a^⁎^	1.72 ± 0.04e	6.98 ± 0.06d	9.58 ± 0.05c	11.49 ± 0.04b	12.44 ± 0.06a
b^⁎^	2.85 ± 0.07e	17.6 ± 0.07d	23.29 ± 0.1c	24.14 ± 0.03b	24.93 ± 0.04a
FT–IR					
1047/1022	0.709 ± 0.006e	0.801 ± 0.011b	0.824 ± 0.012a	0.756 ± 0.008c	0.736 ± 0.006d
1022/995	1.291 ± 0.010a	1.227 ± 0.008b	1.077 ± 0.007d	1.167 ± 0.005c	1.151 ± 0.003c
In vitro digestive parameters					
RDS (%)	39.78 ± 0.22b	32.99 ± 0.59d	32.39 ± 0.1e	37.93 ± 0.07c	45.57 ± 0.12a
SDS (%)	44.56 ± 0.53a	44.34 ± 0.92a	42.54 ± 0.4b	36.37 ± 0.36d	38.21 ± 0.9c
RS (%)	15.67 ± 0.34c	22.67 ± 0.36b	25.07 ± 0.31a	25.7 ± 0.41a	16.22 ± 0.78c
C_∞_ (%)	89.58 ± 3.36b	84.78 ± 2.46c	80.79 ± 1.58e	82.22 ± 3.14d	91.27 ± 3.04a
K (min^−1^)	0.034	0.032	0.029	0.044	0.050
R^2^	0.9812	0.9866	0.9949	0.9668	0.9715

The values represent means of triplicate ± standard deviation. Values in the same column with different letters are significantly different (P < 0.05).

### 3.4 Analysis of texture

Texture is identified as a fundamental factor that influences consumer preference, and high–quality rice noodles are expected to exhibit an optimal balance between hardness and soft elasticity to ensure a pleasant chewing experience. The effect of PCF on the textural properties of rice noodles was systematically evaluated, as detailed in Table 2. Notable increases were observed in hardness, gumminess, and chewiness, whereas cohesion and elasticity significantly decreased as the PCF increased. The increase in hardness ranged from 10.6 % to 32.3 %, whereas the increase in gumminess ranged from 3.2 % to 43.2 %. Similarly, the chewiness increased from 15.9 % to 69.3 %. In contrast, the reduction in cohesiveness ranged from 10.8 % to 29.7 %, and the decrease in springiness was found to fall within the range of 1.2 % to 22.8 %. Hardness is a critical parameter for evaluating rice noodles, because it reflects the force required to achieve a specified level of deformation. The SB value of the pasting characteristics indicated that the PCRN hardness should gradually decreased. However the texture characteristics indicated that the PCRN hardness gradually increased, which may be related to the PCF protein content. Li, Ji, and Li (2022) established a positive correlation between the hardness of rice and its protein content, suggesting that a higher protein content corresponded to increased hardness. The protein content in PCF was found to be significantly higher than that in the rice noodles, which likely contributed to the gradual increase in hardness observed with PCF addition. Furthermore, hardness exhibits a significant negative correlation with cohesion and elasticity (Jia et al., 2023), which is consistent with the experimental results of this study. Additionally, the presence of dietary fibre and lipids in PCF is hypothesised to enhance the texture and firmness of food products (Zheng et al., 2024).

### 3.5 Analysis of Colour

Colour is a critical sensory attribute of food products, significantly influencing consumer acceptance and preference. The PCRN colour variations were analysed, as detailed in Table 2, where L^⁎^ values represent brightness, a^⁎^ values indicate the degree of red or green tones, and b^⁎^ values reflect the presence of blue or yellow tones (Levent & Yuksel, 2022). Significant differences (*P* < 0.05) were observed in the surface colour parameters L^⁎^, a^⁎^, and b^⁎^ between the control group and PCF–treated rice noodles. PCF incorporation resulted in a significant reduction (P < 0.05) in the L* value of PCRN compared to the control group. Specifically, the L^⁎^ value of the rice noodles decreased from an initial value of 97.43 to 77.93 with the addition of 5 % PCF. Conversely, the a^⁎^ and b^⁎^ values progressively increased with higher PCF concentrations. These results indicate that the addition of PCF not only reduces the brightness of PCRN but also enhances the green and yellow tones. Consequently, the colour of the rice noodles shifted from white to yellow as the proportion of PCF increased. This colour transformation was likely attributable to the presence of flavonoids or other pigmented compounds in PCF (Zhao et al., 2018).

### 3.6 Analysis of XRD

**Fig. 1:**
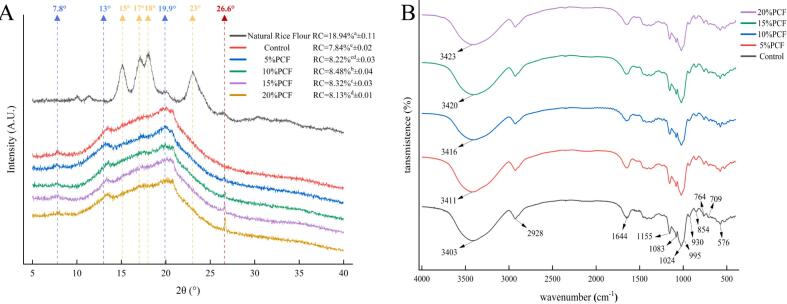
XRD from PCRN and RN in different proportions(A); FT–IR from PCRN and RN in different proportions(B).

Fig. 1A presents the XRD patterns of both natural rice flour and prepared rice noodles. The XRD patterns were utilised to reflect the long–range ordered structure of the starch system, typically indicating the crystalline type and configuration of starch (Chi et al., 2021). The diffraction peaks observed in natural rice flour at 2*θ* angles of 15° and 23°, along with the overlapping double peaks at 17° and 18°, imply that natural rice flour exhibits a predominantly A–type crystalline structure (Zhong et al., 2020). By contrast, the minor diffraction peak detected at 19.9° was potentially associated with the formation of a starch–lipid complex within the rice noodles, which would be consistent with the findings of Kang et al. (2021) and He, Zheng, Wang, Li, and Chen (2020). However, in the XRD patterns of the fresh wet rice noodles produced from natural rice flour, a shift in the positions of the characteristic peaks was observed. Diffraction peaks at 7, 15, and 19.9° indicated that the crystalline type of the rice noodles had transitioned from A–type to V–type. The application of high–temperature and high–humidity heat treatment during processing disrupts the original starch architecture, leading to degradation of the crystalline regions of the starch and alteration of the starch crystal type. Furthermore, compared to natural rice flours, the gelatinization effect causes the diffraction peaks in the rice noodles to become wider and rounder. The characteristic peak positions of rice noodles, which were produced with the incorporation of PCF, remained consistent with those of the control sample, indicating that PCRN maintained its V–type crystal structure. Upon further analysis, a faint crystalline peak was detected at approximately 17°, suggesting the potential formation of a B + V–type starch crystal within the rice noodles. Notably, a distinct characteristic peak was identified at 26.6°, which exhibited a progressive increase in intensity with increasing PCF content. This peak was likely formed by dietary fibre present in PCF(Chen, Lawton, Thompson, & Liu, 2012).

The RC of the samples exhibited significant variation (*P* < 0.05), with natural rice flour demonstrating a markedly higher RC than fresh wet rice noodles. The RC of PCRNs consistently exceeded that of the control rice noodles. This study demonstrates that the RC of starch can reflect the strength of its corresponding crystalline structure. Higher RC values are associated with a stronger crystalline starch structure, resulting in fewer exposed enzymatic sites and reduced susceptibility to enzymatic hydrolysis. Consequently, starch with an elevated RC was able to exhibited enhanced enzymatic resistance. Research conducted by Wang, Li, Copeland, Niu, and Wang (2015) indicated that an increased in crystalline content and the presence of more complete crystalline regions led to higher and more defined diffraction peaks in the samples. Comparable alterations were observed in our experiments, implying that PCF incorporation enhanced the crystalline structure of the starch. The changes in the RC may have been caused by the embedding of dietary fibre from the PCF into the starch matrix. This embedding effect could help maintain the crystallinity of starch, which may be attributed to incomplete gelatinization due to reduced hydration and swelling of raw starch granules in the presence of dietary fibre (Zhang, Sun, & Ai, 2022; Zhang, Wang, et al., 2022). The maximum RC value observed for PCRN was 8.48 % with the incorporation of 10 % PCF. This finding indicated that the addition of 10 % PCF enhanced the stability of the starch crystalline structure while minimising the availability of enzyme-binding sites. Xiao et al. (2021) revealed that the numerous hydrophilic groups in dietary fibres competed with starch molecules for water, thereby diminishing the hydration of starch, reducing the mobility of starch chains, and impeding the rearrangement of starch molecules, ultimately affecting RC values. Another study demonstrated that low concentrations of guar gum integrated into lotus starch effectively maintained the crystallinity and short–range ordered structure of starch gels; conversely, higher concentrations compromised their crystalline integrity (Zheng et al., 2019). Moreover, the substantial addition of the PCF resulted in a dilution of starch granule concentration, which further limited the rearrangement of starch molecules, thereby contributing to a reduction in RC (Ren, Linter, & Foster, 2020). In addition, branched starches, which participate in the formation of crystalline regions, may lead to the development of amorphous zones in the presence of dietary fibre. The accumulation of this amorphous phase may obscure contact sites for digestive enzymes, consequently inhibiting the starch digestion.

### 3.7 Analysis of FT–IR

Fig. 1B shows the FT–IR spectrum of PCRN, which displayed characteristic absorption features associated with starch. The absorption peaks observed at 1155, 1083, and 1024 cm^−1^ were attributed to the C—O—C stretching vibrations inherent to starch. Additionally, the peaks identified at 995, 930, 854, 764, 709, and 576 cm^−1^ corresponded to the stretching vibrations of the starch backbone (Han et al., 2020). The peak at 2928 cm^−1^ was associated with the C—H stretching vibration, while the peak at 1644 cm^−1^ represented the C—O—O stretching vibration within the carbohydrate structure (Li, Jiang, et al., 2023; Li, Li, et al., 2023; Li, Shen, et al., 2023； Liu et al., 2023). The prominent and broad absorption peaks observed around 3400 cm^−1^ in rice noodles were primarily ascribed to the stretching vibrational absorption of O—H bonds. This phenomenon suggests the presence of hydroxyl groups in the starch that participate in either intramolecular or intermolecular hydrogen bonding. Notably, the absorption peaks of PCRN in this spectral region exhibited varying degrees of shift, resulting in a broader band spanning from 3700 to 3000 cm^−1^, which served as a significant indicator for evaluating the strength of hydrogen bonding (Yang et al., 2024). Comparative analysis of the FT–IR spectra of PCRN revealed that no new infrared absorption peaks were generated, even with an increased proportion of PCF. This observation implies that covalent bond structures were not formed between PCF and the rice noodles; rather, their interaction was mediated through noncovalent bonds, such as hydrogen bonding.

The intensity ratios of the three characteristic peaks observed in the infrared spectra served to quantify the ordered and disordered structures present within the short–range architecture of starch. Specifically, the absorption peak at 1047 cm^−1^ was indicative of the ordered structure of the starch, whereas the peak at 1022 cm^−1^ was associated with the structural attributes of the amorphous region. Furthermore, the absorption peak at 995 cm^−1^ pertained to the hydrogen bonding interactions among the starch molecules. Collectively, these spectral features offered significant insights into the structural properties of starch (Hong, Zhang, Xu, Wu, & Xu, 2021；Man et al., 2012). The 1047/1022 ratio served as an indicator of the degree of molecular order within the starch, with higher values reflecting a more organised molecular arrangement. By contrast, the ratio of 1022/995 was utilised to assess the extent of amorphous structural regions present in the starch; an elevated ratio signified a greater proportion of amorphous regions and a more disordered short–range structure (Zhang, Fan, Xie, Zhang, & Fu, 2023). As illustrated in Table 2, the ratio of 1047/1022 initially rose and subsequently declined with increasing PCF levels. In contrast, the ratio of 1022/995 exhibited a decreasing trend, followed by an increase as PCF levels increased. These observed patterns suggested that the incorporation of PCF significantly enhanced (*P* < 0.05) the degree of short–range ordering in the PCRN. Previous studies have indicated that a high degree of short–range ordering is effective in resisting enzymatic hydrolysis (Toutounji et al., 2019). Consequently, the enhancement of short–range ordering, attributed to the addition of PCF, contributed to the improved enzymatic resistance of PCRN.

### 3.8 Analysis of SEM

**Fig. 2:**
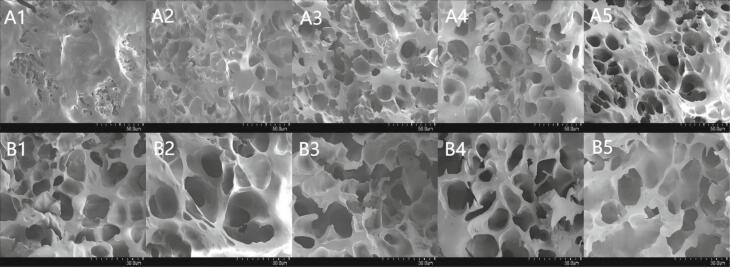
SEM from PCRN and RN in different proportions. A: x800; B: x1500; 1: Control; 2: 5 %; 3: 10 %; 4: 15 %; 5: 20 %.

Fig. 2 shows the SEM images of the rice noodles. Fig. 2A and Fig. 2B present the cross–sectional morphology of freeze–dried rice noodles at 800× and 1500× magnification, respectively. Panels 1–5 show SEM images of the control group and PCRN samples with varying concentrations of PCF. Analysis of Fig. 2A reveals that the cross–section of the rice noodles in the control group exhibited a relatively smooth texture. An increase in the amount of PCF was correlated with a continuous increase in the number of pores within the PCRN, accompanied by a discernible trend toward larger pore sizes. This observation may be attributed to the effects of cellulose, hemicellulose, and lignin present in PCF on the starch gelation process occurring in rice noodles (Zheng et al., 2024). Therefore, in the control group, the high starch content and increased degree of gelatinization facilitated significant cross–linking among the starch granules, resulting in the development of a smoother surface. At a magnification of 1500×, the rice noodles displayed a pronounced honeycomb network structure, corroborating the observations made of Liu et al. (2021). As the PCF concentration increased, a well–defined lamellar structure progressively emerged within the rice noodles, accompanied by the formation of larger expansion bubbles. The protein content of the PCF surpassed that of natural rice flour, thereby promoting the establishment of more effective protein–starch cross–linking structures. This enhancement effectively mitigates starch swelling, leading to a denser starch network structure, which is advantageous for inhibit the digestion rate of rice noodle. In addition, PCF is rich in active compounds, including polysaccharides and polyphenols. Existing research suggests that these compounds could interact with starch granules and adhere to their surfaces. Through the intertwining process, the interaction between starch granules and polysaccharides results in a tighter and more organised microstructure, as highlighted by Ren, Linter, and Foster (2020) and Ren et al. (2020). Chen et al. (2019) demonstrated that polysaccharide molecules within a gel system adhere to the surface of starch granules *via* hydrogen bonding and other non–covalent interactions. The formation of a dense network structure between starch granules, dietary fibres, proteins, and additional components within the PCF matrix facilitated the encapsulation of loose starch granules, thereby diminishing the interaction between enzymes and starch. This encapsulation effect is advantageous for reducing the digestibility of starch. However, when the proportion of PCF exceeded 10 %, a slight reduction in size of expansion bubbles was observed, accompanied by a noticeable thickening of the layer structure between the starch granules. This reduction was attributed to an excess of dietary fibre, which disrupted the protein–starch cross–linking structure, resulting in starch overflow and inhibiting the network structure from completely encapsulating all starch granules. This disruption led to direct enzyme–starch contact, facilitating rapid starch digestion.

### 3.9 Analysis of CLSM

**Fig. 3:**
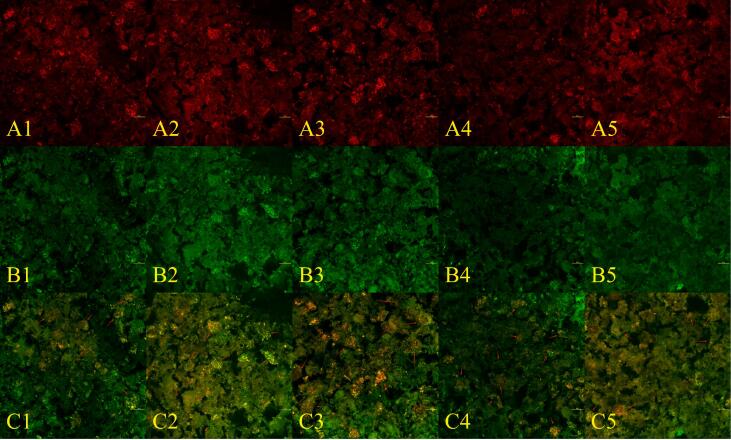
CLSM from PCRN and RN in different proportions. (A): Protein network structure; (B): Starch structure; (C): Complex structures of protein networks and starch; (1): Control; (2): 5 %PCF; (3): 10 %PCF; (4): 15 %PCF; (5): 20 %PCF; All images were captured at 2048 × 2048 resolution.

Fig. 3 shows the CLSM images of PCRN with different amounts levels of PCF. In these images, the protein component was stained red, the starch component was green, and the co–localised regions of both components appeared yellow, as marked by red arrows (Jia, Ma, & Hu, 2020). Fig. 3A shows a single–channel CLSM image of the protein components. Owing to the higher protein content in PCF than in natural rice flour, a gradual increase in the stained protein fraction was observed with increasing levels of PCF incorporation. In Fig. 3C, a two–channel stacked plot, the yellow region of the sample was observed to expand with the incorporation of PCF, suggesting that the protein–starch network structure of rice noodles became more compact with appropriate levels of PCF. The observed increase in the yellow area further indicates a notable enhancement in the network structure, consistent with our hypotheses derived from the SEM findings. A robust protein–starch spatial network structure was more adept at encapsulating starch, thereby minimising its exposure and reducing the availability of enzyme contact sites, which enhanced the starch's resistance of starch to digestion, a conclusion supported by subsequent digestion experiments. However, when the addition of PCF exceeded 10 %, Fig. 3C showed that the area of the yellow region was significantly reduced compared with that of the sample containing 10 % PCF, and the larger network structure observed in the 10 % sample was not replicated. This phenomenon was attributed to the increase in PCF, which contained substantial dietary fibre (as documented in the Materials section). Consequently, elevated PCF content directly increased the dietary fibre concentration within the rice noodles. Firstly, the incorporated fibre compromised the native protein–starch network architecture, manifesting as diminished yellow–stained areas under CLSM analysis. Secondly, this structurally compromised network forfeited its starch–encapsulating capacity, permitting starch leakage. This masked the protein network structure, making it unobservable. Ultimately, these microstructural changes likely contributed to the attenuated digestion resistance observed in higher–PCF formulations.

### 3.10 Analysis of *in vitro* digestibility

**Fig. 4:**
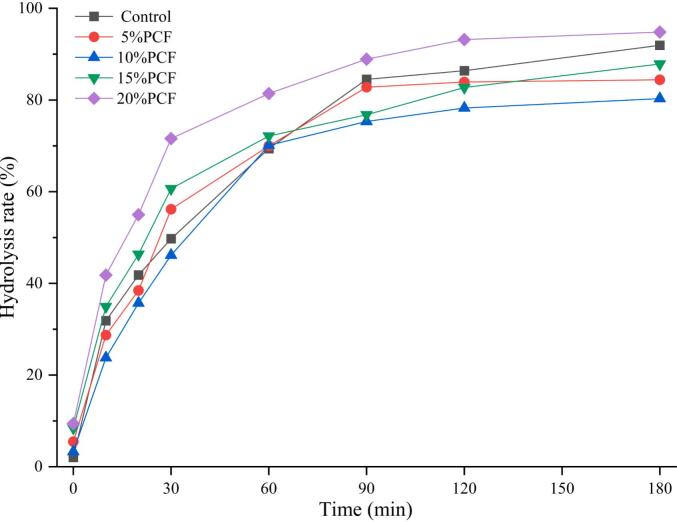
*In vitro* hydrolysis profiles of starches from PCRN in different proportions.

The crystalline structure of starch is irreversibly damaged during pasting, which subsequently modifies the nutritional characteristics of starchy foods (Li, 2022). As shown in Fig. 4, the rate of starch hydrolysis in each experimental group exhibited rapidly increased within the initial 30 min. The incorporation of PCF altered the digestive properties of rice noodles. After a digestion period of 180 min, the degree of starch hydrolysis was ranked as follows: 20 % (94.83 %) > control (91.95 %) > 15 % (87.86 %) > 5 % (84.42 %) > 10 % (80.31 %). These findings suggested that PCF exerts an inhibitory effect on rice noodle digestibility, which is consistent with our previous experimental observations. Notably, starch hydrolysis increased significantly when the concentration of PCF exceeded 10 %, supporting our earlier hypotheses based on the SEM and CLSM results. Starch digestibility is a critical parameter for evaluating the quality of starchy foods because it is strongly associated with variations in blood glucose levels following human consumption (Goñi, Garcia-Alonso, & Saura-Calixto, 1997). Starch can be categorised into three distinct types based on its digestibility: RDS, SDS, and RS. RDS is characterised as starch that is digested within 20 min, SDS is starch that is digested over 20 to 120 min, and RS remains starch that undigested after 180 min. Table 2 illustrates the proportions of these three starch types present in PCRN. In the control group, the percentages of RDS, SDS, and RS were recorded at 39.78 %, 44.56 %, and 15.67 %, respectively. Notably, the RS content in rice noodles with 10 % PCF increased to 25.07 %, significantly higher than that observed in the control group. In contrast, the RDS content decreased to 32.39 %. The variations in the three starch types in the samples with different amounts of PCF addition also demonstrated different degrees of change.

**Fig. 5:**
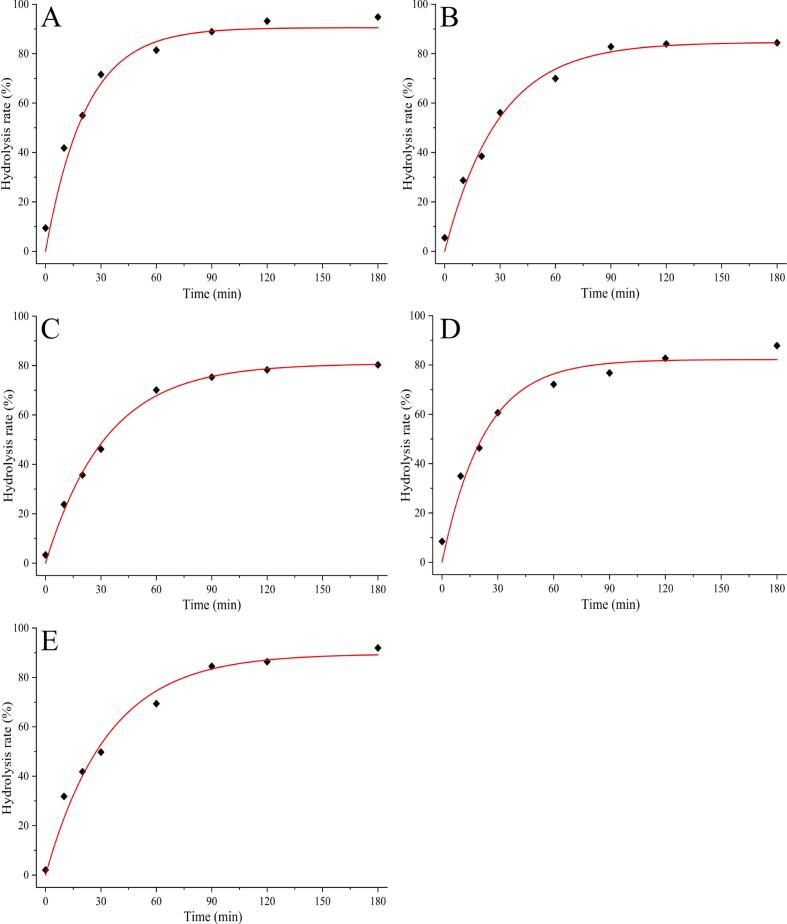
Kinetic model of starch hydrolysis from PCRN in different proportions. A: Control; B: 5 %PCF; C:10 %PCF; D: 15 %PCF; E: 20 %PCF.

The starch hydrolysis curve conforms to a first–order kinetic model, which facilitates the representation of the digestion curve through the eq. C = C_∞_ (1 – e^–kt^). In this equation, C signifies the rate of starch hydrolysis (in %), C_∞_ represents the equilibrium percentage achieved after 180 min, and k denotes the kinetic constant associated with the hydrolysis reaction rate (in min^−1^) (Zhang et al., 2018). The parameter C_∞_ indicates the final equilibrium point of digestion, while the value of k provides insight into the rate of this equilibrium process. Thus, lower values of C_∞_ and k suggested a higher content of RS and a reduced rate of starch hydrolysis in the analysed samples. R^2^ indicates the quality of the curve fitting. The detailed parameters and the corresponding fitted curves are shown in Fig. 5 and Table 2, respectively. The fitted digestive hydrolysis curves for PCRN with additions of 0 % – 20 % are shown from A to E in Fig. 5. Notably, the k value for PCRN, with a 10 % addition of PCF was lower than that of the other rice noodle samples. This finding suggests that the incorporation of 10 % PCF was optimal to achieve the desired low–hydrolysis characteristics.

### 3.11 Analysis of antioxidant analysis

**Fig. 6:**
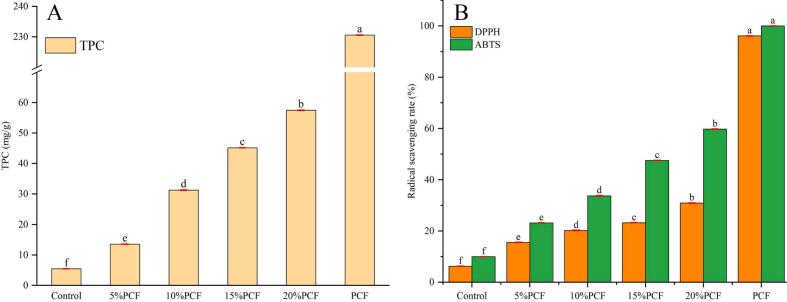
Total Phenolic Content from PCRN and RN in different proportions (A); Antioxidant Capacity from PCRN and RN in different proportions (B).

Fig. 6 presents the TPC and antioxidant properties of the PCF and PCRN. Recent research has indicated that polyphenols serve as potent antioxidants that can mitigate the risk of several chronic diseases, such as tumours, chronic inflammation, and cardiovascular disorders (Abd El-Hack et al., 2023). Chen, Yang, and Chen (2022) analysed the main antioxidant active constituents in PCF and isolated five major compounds: 5–carboxymethylfurfural, scopolamine, isoquercetin, hyperoside and rutin. The TPC values of each sample are illustrated in Fig. 6A. The TPC of the PCF sample was significantly higher than those of the other samples. This enhancement can be attributed to the elevated polyphenol concentrations in the PCF. Consequently, the TPC of the samples increased with the incorporation of PCF to levels exceeding that of the control rice noodles. This finding indicated that a significant amount of phenolic compounds was retained in PCRN, demonstrating a dose–dependent effect that played a decisive role in enhancing the rice noodle antioxidant activity. Fig. 6B shows the assessment of antioxidant capacity using both DPPH and ABTS assays. Antioxidants neutralized free radicals primarily through two mechanisms: electron transfer reduction and hydrogen atom transfer (Zhang et al., 2023). As expected, the radical scavenging capacity (ABTS and DPPH) of the PCRN samples was significantly higher than that of the control rice noodles. The DPPH and ABTS scavenging rates increased from 6.3 % and 11.25 % to the peak values of 30.94 % and 60.54 %, respectively, with growth rates ranging from 146.98 % to 391.11 % (DPPH) and from 97.54 % to 438.19 % (ABTS). Additionally, other bioactive components present in PCF, such as polysaccharides, contribute to the enhanced antioxidant capacity of PCRN. Consequently, it can be inferred that the incorporation of PCF not only significantly enhanced the antioxidant capacity but also preserved a considerable amount of bioactive compounds in rice noodles.

### 3.12 Sensory evaluation of PCRN

Fig. S1A shows the results of the sensory evaluation of the rice noodle samples, indicating that their overall quality was within the acceptable range, and the overall rice noodle sensory evaluation scores were all above 70 points (Liu & Wang, 2019). Among these, the sensory evaluation of PCRN with a 10 % added amount was the highest. Fig. S1B shows the scores for each evaluation indicator. The aroma value of rice noodles increased with an increase in PCF content, possibly owing to the high content of phenolic substances in PCF, which helps to form a better flavour in rice noodles. The surface structure, texture characteristics, and taste of the rice noodles had the highest scores when the addition amount was 10 %, which agreed with the texture results. These results showed that the addition of PCF had a significantly impacted the quality of rice noodles and that an appropriate amount of PCF could improve rice noodle quality and flavour.

## 4 Conclusion

This study investigated the effects of varying amounts of PCF on the pasting and thermal properties of compound powders, as well as the cooking quality, texture, colour, microstructure, *in vitro* digestion ability, antioxidant capacity, and sensory evaluation of rice noodles. The results indicated that PCF reduced the viscosity of the compound powder, effectively inhibited the ageing of rice flours, and increased the ΔH. The addition of PCF significantly altered the appearance and texture of rice noodles, leading to increased hardness, a yellow hue, and the formation of more pores within the noodles. Incorporating an appropriate level of PCF into rice noodles could enhance the microstructure of starch, inhibit starch digestion, and consequently lower starch digestibility. Additionally, the inclusion of PCF could augmented the active substances in rice noodles, improved their antioxidant capacity, enriched their nutritional content, and enhance their overall nutritional value. After comprehensive analysis, a 10 % addition of PCF was deemed optimal. This study suggests that the incorporation of PCF into rice noodles could produce nutrient–dense products while reducing digestibility.

## CRediT authorship contribution statement

**Yongguang Zhu:** Writing – original draft, Visualization, Validation, Methodology, Investigation, Formal analysis, Data curation, Conceptualization. **Qianwei Cheng:** Supervision. **Jiayan Zhang:** Methodology, Conceptualization. **Peng Chen:** Supervision, Conceptualization. **Yuan Cheng:** Supervision, Conceptualization. **Luli Meng:** Writing – review & editing, Supervision, Methodology, Conceptualization. **Jie Chen:** Investigation, Conceptualization. **Tong Chen:** Supervision, Methodology, Conceptualization.

## Declaration of competing interest

The authors declare that they have no known competing financial interests or personal relationships that could have appeared to influence the work reported in this paper.

## Data Availability

Data will be made available on request.

## Appendix A Supplementary data

Supplementary material Image 1
