# Supplementary material for: Effects of Polygonatum cyrtonema flour addition on the food quality, microstructure, in vitro digestibility and antioxidant capacity of rice noodles
Source: Food Chem X. 2025 Jul 3;29:102710. doi: 10.1016/j.fochx.2025.102710 (PMC12272932; doi:10.1016/j.fochx.2025.102710)
Supplement: Supplementary file 1 — Supplementary material [file mmc1.docx]

**Table S1**

The table of sensory evaluation

| Scoring items | | Scoring Criteria | Score |
| --- | --- | --- | --- |
| Aroma  (0 ~ 15) | Flavor  (15) | Smells of both rice aroma and strong special flavour | 11~15 |
|  |  | Smells of both rice aroma and slight special flavour | 6~10 |
|  |  | Only rice aroma or some odor flavour | 0~5 |
| Surface structure  (0 ~ 25) | Color  (6) | No flecks and no mixed colors | 5~6 |
|  |  | Have some stray colors | 3~4 |
|  |  | Strange color | 0~2 |
|  | Glossy  (5) | Appropriate glossy | 4~5 |
|  |  | Light glossy | 2~3 |
|  |  | No glossy | 0~1 |
|  | Structural integrity  (8) | Tightly structured, no broken or crumbled strips | 7~8 |
|  |  | Slightly easy to be broken, exist a few broken pieces | 5~6 |
|  |  | Easy to be broken, exist lots of broken pieces | 0~4 |
|  | Evenness  (6) | Smooth surface and uniform thickness | 5~6 |
|  |  | Slightly rough surface and slightly uneven thickness | 3~4 |
|  |  | Rough surface and uneven thickness | 0~2 |
| Texture properties (0 ~ 45) | Viscosity  (15) | Appropriately sticky, non-stick teeth | 11~15 |
|  |  | Slightly stick to teeth | 6~10 |
|  |  | Strongly stick to teeth | 0~5 |
|  | Softness  (15) | Medium soft and medium hard | 11~15 |
|  |  | Slightly soft or slightly hard | 6~10 |
|  |  | Very soft or very hard | 0~5 |
|  | Tenderness  (15) | Appropriately chewy | 11~15 |
|  |  | Slightly chewy or slight non-chewy | 6~10 |
|  |  | Very chewy or very non-chewy | 0~5 |
| Taste  （0 ~ 15） | Fresh wet rice noodle taste | Tastes great and totally acceptable | 12-15 |
|  |  | Tastes fine and acceptable | 8-11 |
|  |  | Tastes average and barely acceptable | 5-8 |
|  |  | Tastes bad and unacceptable | 0-4 |





**Fig. S1.** Sensory quality from PCRN and RN in different proportions. A: sensory evaluation total points; B: sensory evaluation radar chart
